# UPLC-PDA determination of paeoniflorin in rat plasma following the oral administration of Radix Paeoniae Alba and its effects on rats with collagen-induced arthritis

**DOI:** 10.3892/etm.2013.1358

**Published:** 2013-10-23

**Authors:** DAN WU, JIANG CHEN, HAO ZHU, XIN-GUI XIONG, QING-HUA LIANG, YANG ZHANG, YONG ZHANG, YANG WANG, BO YANG, XI HUANG

**Affiliations:** 1 Department of Traditional Chinese Medicine, Central South University, Changsha, Hunan 410008, P.R. China; 2 Center of Telemedicine, Xiangya Hospital, Central South University, Changsha, Hunan 410008, P.R. China

**Keywords:** ultra performance liquid chromatography, Radix Paeoniae Alba, paeoniflorin, collagen-induced arthritis, rheumatoid arthritis, bizhongxiao decoction

## Abstract

Rheumatoid arthritis (RA) is a chronic disabling autoimmune disease with characteristics of chronic, progressive inflammatory joint synovial damage, which mainly encroaches upon the synovium of the joint. The use of traditional medicine to treat RA slows the development of RA to a certain extent; however, it often has numerous side-effects. Therefore, the focus of RA research is the identification of a new, safe and effective medicine. The aim of the present study was to use an ultra performance liquid chromatography and photo diode array (UPLC-PDA) method to detect the paeoniflorin component in a Radix Paeoniae Alba decoction and in rat plasma following the oral administration of Radix Paeoniae Alba decoction. In addition, the effects of paeoniflorin on collagen-induced arthritis (CIA) in rats were investigated. The results indicate that a UPLC-PDA method for determining the presence of paeoniflorin in the Radix Paeoniae Alba decoction was successfully established. The method was fast, simple, sensitive, precise and valid. Paeoniflorin was shown to be a bioactive component of the Radix Paeoniae Alba decoction that was absorbed into rat plasma. Paeoniflorin significantly improved the disease resistant ability of RA rats and reduced the levels of the inflammatory cytokines, IL-1β and TNF-α, thereby inhibiting inflammation and bone erosion in the rats with CIA. The observations are likely to lay the foundation for further study of the mechanism of paeoniflorin in the treatment of RA.

## Introduction

Rheumatoid arthritis (RA) is a chronic disabling autoimmune disease that causes chronic, progressive inflammatory joint synovial damage, which largely encroaches upon the synovium of the joint. The pathological changes include synovial cell hyperplasia, expansion, congestion, hypertrophy of vessel walls, inflammatory cell infiltration, fibrous tissue hyperplasia, transparency and degeneration (1). The primary goal of RA therapy is to control inflammation and joint erosion. The pathogenesis of RA has not been fully elucidated, but may be associated with the immune system and inflammatory reaction. Traditional medicine may slow the development of RA to a certain extent; however, numerous side-effects are often observed. Therefore, the focus of RA research is the identification of a safe and effective medicine.

Bizhongxiao decoction (BZXD) is a traditional Chinese medicine (TCM), which was formulated by the Department of Traditional Chinese Medicine (Changsha, China). The medicine has been used clinically to treat RA for a number of years and has demonstrated good clinical efficacy (2). The reported actions of BZXD include the regulation of the immune system, the inhibition of inflammatory cytokines, synovial angiogenesis and bone destruction, and the modulation of the abnormal expression of genes and proteins (3–8). However, since BZXD is a constituent of numerous TCMs and has a complex chemical composition, the comprehensive study of its mechanism of action in the treatment of RA is limited. Radix Paeoniae Alba is an important component of BZXD. It is obtained from a type of peony and is slightly cold, bitter and sour, with efficacy in calming the liver, relieving pain, nourishing menstruation, astringing Yin and hidroschesis (9). Medicinal Radix Paeoniae Alba is the dried root of the Ranunculaceae plant, *Paeonia lactiflora* Pall, from which the skin has been removed (10). Previous pharmacological studies of Radix Paeoniae Alba have shown that it has anti-inflammatory, analgesic, antispasmodic, liver protection and immune regulatory functions (11). The effective components of Radix Paeoniae Alba are mainly composed of a series of aminoglycoside substances, including paeoniflorin, hydroxy-paeoniflorin, peony glucoside, albiflorin and benzoylpaeoniflorin, which are collectively referred to as the total glucosides of peony (TGP). Paeoniflorin accounts for >90% of the total glucosides in Radix Paeoniae Alba and is the main effective component. Paeoniflorin has been found to mediate a wide range of pharmacological effects, including hypoglycemic, antitumor, immunomodulatory, anti-inflammatory and neuronal protection actions (12). One study demonstrated the ability of paeoniflorin to inhibit the generation of interleukin-1 (IL-1), tumor necrosis factor-α (TNF-α) and PGE2 in peritoneal macrophages in rats with adjuvant arthritis (AA) (13). In addition, orally administered paeoniflorin has been shown to significantly reduce paw edema in rats with collagen-induced arthritis (CIA), thereby improving the inflammation of multiple joints (14).

At present, the use of the ultra performance liquid chromatography and photo diode array (UPLC-PDA) method to determine the paeoniflorin composition in Radix Paeoniae Alba decoction, and in plasma following the intragastric administration of Radix Paeoniae Alba decoction to rats, is rarely reported in the literature. However, the present study used the UPLC-PDA method for this purpose and also explored the therapeutic effect of paeoniflorin when administered to rats with CIA. The aim of the study was to lay the foundations for further studies of the mechanism of paeoniflorin and the TCM, BZXD, in the treatment of RA.

## Materials and methods

### UPLC-PDA analysis of paeoniflorin in Radix Paeoniae Alba decoction and in rat plasma following the oral administration of Radix Paeoniae Alba decoction

#### Preparation of drugs and standards

Radix Paeoniae Alba was purchased from the Xiangya Hospital of Central South University (Changsha, China). It passed identification by the Research Institute for Pharmacology of Traditional Chinese Medicine of Xiangya Hospital, Central South University. Radix Paeoniae Alba was crushed into powder and then pure water was added in the ratio of 1:8 of powder to water. The aqueous composition was boiled for 30 min, filtered to obtain the liquid and then rotary evaporated at 60°C and low pressure to provide a concentrated aqueous solution containing only one traditional Chinese medicine. A freeze dryer was used to transform the concentrate into a freeze-dried powder, with a yield of 18.5%. The powder was sealed and stored at 4°C. A reference substance of paeoniflorin was purchased from The National Institute For The Control of Pharmaceutical and Biological Products (Beijing, China) and the mass fraction was >98%.

#### Chromatographic conditions

UPLC was performed using an Acquity UPLC system (Waters Corporation, Milford, MA, USA), which included a binary pump processor, sample processor, column oven, PDA detector and Empower chromatography workstation. The mobile phase consisted of acetonitrile and 1% acetic acid in the ratio 22:78 under the following conditions: Detection wavelength, 190–480 nm; flow rate, 0.25 ml/min; column temperature, 40°C; and injection volume, 5 μl. The analysis time was 4 min. The number of theoretical plates was calculated using the paeoniflorin peak and was not <5,000. Acetic acid, acetonitrile and methanol were AR grade and self-prepared triple-distilled water was used.

#### Preparation of the reference substance solution

Paeoniflorin was weighed to 0.41 mg accurately, put into a 10-ml brown volumetric flask and methanol was added for ultrasonic dissolution. The solution was diluted to scale and shaken. A paeoniflorin reference stock solution was obtained with a concentration of 0.041 mg/ml. The reference solution was sealed and stored at 4°C for later use.

#### Preparation of the test solution

Radix Paeoniae Alba freeze-dried powder was weighed accurately to 5 g with a 1% electronic balance (equivalent to 27.03 g crude drug). The powder was ultrasonically dissolved in 200 ml water for 10 min and Radix Paeoniae Alba decoction, with a concentration of 0.135 g crude drug/ml, was obtained. Next, 2 ml decoction was measured accurately, placed into a 10-ml volumetric flask and 7 ml methanol was added followed by 30 min ultrasonic oscillation. After maintaining at room temperature for 30 min, 7 ml methanol was added with shaking and the resultant mixture was filtered. The filtrate was filtered using a 0.45-μm membrane and the test solution comprising Radix Paeoniae alba (0.027 g crude drug/ml) was obtained.

#### Preparation of the plasma sample

Experiments were performed using male Sprague Dawley (SD) rats (weight, 200–220 g) provided by the Animal Experimental Center of the Hunan People’s Hospital (Changsha, China). All experiments conformed to the Regulations for the Administration of Affairs Concerning Experimental Animals (1988) and were approved by the Animal Experimental Center for Central South University. Normal SD rats were divided into a Radix Paeoniae Alba gastric perfusion group and a blank control group, and were fasted for 12 h. An oral decoction of Radix Paeoniae Alba was administered to the Radix Paeoniae Alba group at a dose of 1.35 g/kg crude drug/body weight (converted according to the surface area of a 70 kg human) (15). To the blank control group was administered a dose of double-distilled water by gavage. After 30 min, the rats were sacrificed and blood samples were collected in anticoagulant tubes. The samples were allowed to stand at room temperature for 2 h, prior to centrifugation at 1,000 × g for 15 min, in order to obtain the rat plasma. Following this, 2 ml ethyl acetate, 4 ml acetonitrile and 1.2 ml acetone was added to 2 ml rat plasma and the resulting mixture was irradiated with ultrasound for 20 min. Next, the solutions were centrifuged at 3,000 rpm for 20 min, the supernatant was obtained and placed in a water bath at room temperature. The supernatant was dried with nitrogen, redissolved in 50 μl acetic acid solution (20%) and 50 μl methanol, then centrifuged at 15,000 × g for 20 min. The resulting supernatant was the required plasma sample.

### Screening of the gavage dose of paeoniflorin

#### Animals

The Animal Experimental Center of the Hunan People’s Hospital provided 25 male and female healthy SD rats (clean grade; age, 45–50 days; weight, 150–180 g). Rats had access to food *ad libitum* and were maintained in a 12/12 h light/dark cycle (light time, 6:00–18:00). Background noise was maintained at 40±10 db and the temperature was 20±3°C. The rats were acclimatized to these conditions for 1 week.

#### Replication of the CIA rat model

Following the instructions provided with bovine II-type collagen (BIIC; immunization grade; lot, 120197; Chondrex, Inc., Redmond, WA, USA), replicated CIA models were constructed for 20 rats randomly selected from the 25 SD rats. Firstly, 10 mg BIIC was completely mixed with 5 ml acetic acid (0.05 M) to form a 2 mg/ml BIIC solution. Next, 5 ml of this solution was mixed with 5 ml complete Freund’s adjuvant (070M8704; Sigma-Aldrich, St. Louis, MO, USA), to produce a 1 mg/ml BIIC solution. This BIIC solution (0.2 ml) was then subcutaneously injected into the tail root of each rat for immunization. After 7 days, 5 ml BIIC and 5 ml incomplete Freund’s adjuvant (101M8711; Sigma-Aldrich) were mixed using the same method to prepare 1 mg/ml BIIC solution and a subcutaneous injection of the BIIC solution (0.1 ml) was administered at the tail root of each rat for reimmunization.

#### Animal grouping

Two weeks following the initial injection for immunization, the animal models were randomly divided into low, middle and high dose groups for paeoniflorin treatment. The model control group (model group) and the normal control group (normal group) were also established.

#### Delivery methods

Following immunization (14 days), the treatment groups were treated with the paeoniflorin standards. The administration dosage for the rats was converted according to the surface area of a 70 kg human body. The concentrations of the paeoniflorin standards for the low, middle and high dose groups were 0.5, 1 and 2 mg/kg/day, respectively. The model and normal group rats were able to drink water freely.

#### Detection of serum inflammatory cytokines in the rats

On day 42 following immunization, the rats were fasted for 12 h and then sacrificed. Blood samples were collected and left at room temperature for 2 h. Centrifugation at 111 × g for 15 min was used to obtain the required serum. An ELISA kit (lot, G12030317; Wuhan Huamei Biotech Co., Ltd, Wuhan, China) was used to test IL-1β and TNF-α levels.

### Effect of paeoniflorin on CIA model rats

#### Animals and replication of the CIA rat model

The animal experimental center at the Hunan People’s Hospital provided 60 male and female healthy SD rats (age, 45–50 days; weight, 150–180 g). Rats were maintained as described previously. A total of 40 SD rats were selected for replication of the model, using the modeling methods described earlier.

#### Animal grouping and delivery method

Two weeks following immunization, the animal models were randomly divided into the paeoniflorin treatment (PF group), model and normal groups. According to the screened gavage dose, the concentration of the paeoniflorin standard was 1 mg/kg/day. The model and normal groups were able to drink water freely.

#### Weight and growth of the rats

Body weight was measured on days 7, 14, 21, 28, 35 and 42 following immunization in each group. The increase in body weight was calculated by subtracting the previous weight for every week. General observations of the rats were also recorded, including the mental state, hair quality, diet and activity of the rats.

#### Joint symptom score

A joint symptom score was calculated using the arthritis index (AI) integral method (16). The AI is an objective index, reflecting the occurrence and development of arthritis. Joint redness, the extent and degree of swelling and the joint deformation of rats with arthritic disease were analyzed to determine a grade of between 0 and 4 (0, no arthritis; 1, mild swelling of joints following redness; 2, moderate swelling; 3, severe joint swelling; and 4, severe joint swelling and the inability to be loaded). Higher arthritis exponential integrals represent joint symptoms of greater severity.

#### Measurement of the degree of paw swelling

Every week following immunization, the thickness of the right rear foot of each rat was measured in a fixed position using a fine angle compass and millimeter ruler.

#### Joint synovial histopathology [hematoxylin and eosin (H&E) staining]

On days 14, 28 and 42 following immunization, the rats were sacrificed by cervical dislocation (using anesthesia 10% chloral hydrate) and blood samples were collected. The bilateral knee joint and the whole rear paw, including the ankle joint, were removed. The fur and muscle fiber were removed and the paw was fixed in 10% neutral formalin for 24 h. This was followed by decalcification in 14% EDTA decalcifying fluid for 5 days and neutralization in 5% sodium thiosulfate for 3 h. The samples were washed for 12 h, embedded with dehydrate paraffin and cut into 5–6-μm sections (longitudinal). Sections were placed in a 60°C oven for 30 min, then soaked with xylene twice for 20 min. Next, sections were soaked with 95% ethanol for 3 min and then 80% ethanol for 1 min, washed with distilled water for 1 min, stained with hematoxylin for 15 min and washed. Acid alcohol was used for differentiation for 3 sec, followed by washing with tap water for 10 min. Eosin solution was then used to stain the sections for 3 min, followed by washing. Next, 80% ethanol, 95% ethanol and ethanol were used successively for gradient dehydration. The samples were mounted with a neutral gum and the pathological changes were observed using a light microscope (CX21; Olympus, Tokyo, Japan).

#### Joint X-ray imaging

The presence of joint destruction was investigated in the rat joints after modeling. An intraperitoneal injection of 10% chloral hydrate (3.5 ml/kg) was administered to the rats for anesthesia. The joints of the rat limbs were stretched as far as possible and placed in an X-ray machine for image data collection.

#### Detection of serum inflammatory cytokines

On days 28 and 42, the levels of serum inflammatory cytokines were measured as described earlier.

#### Statistical Analysis

SPSS 19.0 software was used for statistical analysis (SPSS, Inc., Chicago, IL, USA). All data are expressed as the mean ± SD. Group comparison adopts analysis of variance. Prior to analysis of variance, the data had already passed the variance homogeneity and normality tests. Each time point index difference in the group was analyzed by the analysis of variance of repeated measurement design. P<0.05 was considered to indicate a statistically significant result.

## Results

### Paeoniflorin composition

**Figure 1:**
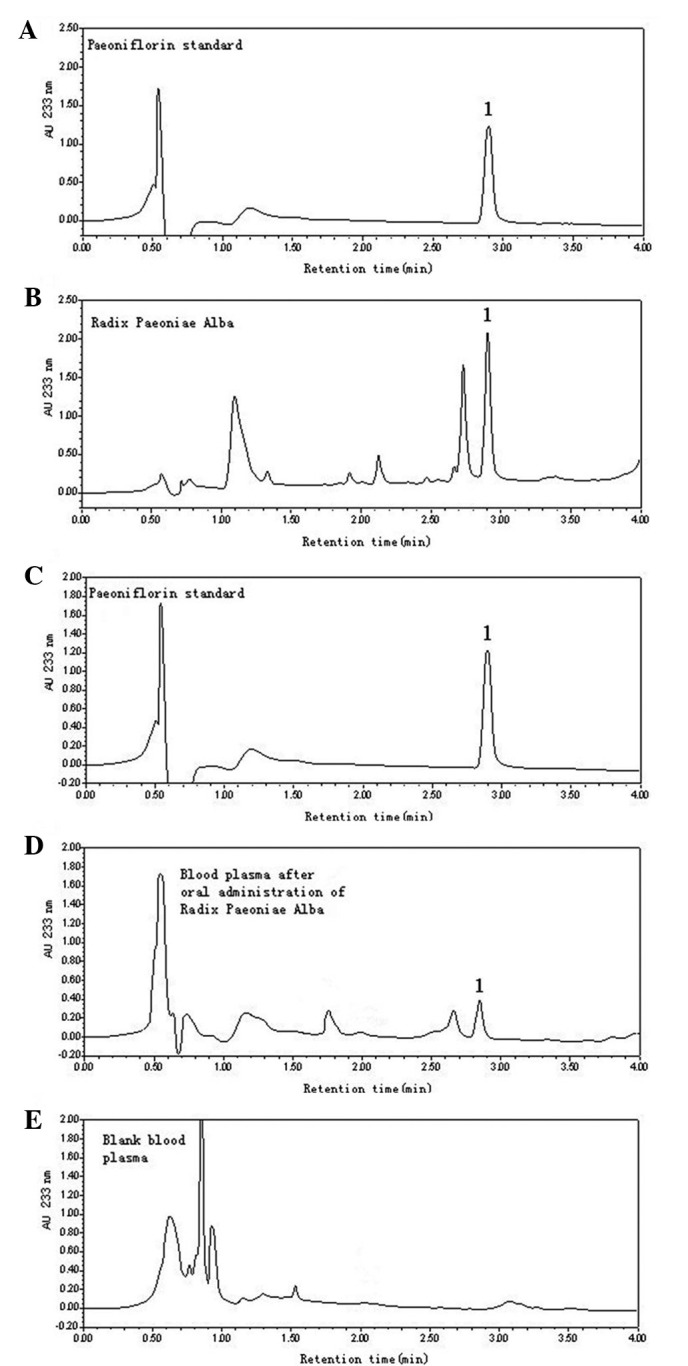
Typical chromatograms at 233 nm of (A and C) the paeoniflorin standard, (B) Radix Paeoniae Alba, (D) the paeoniflorin compound in blood plasma 30 min after the oral administration of Radix Paeoniae Alba decoction to rats and (E) blank blood plasma. The peak labeled 1 represents peoniflorin.

UPLC-PDA was performed to detect the presence of paeoniflorin in the Radix Paeoniae Alba decoction and in the plasma of rats following Radix Paeoniae Alba gavage. Following a number of preliminary experiments, the optimum chromatographic conditions for the separation of paeoniflorin were selected. Using the UPLC-PDA method, under the selected chromatographic conditions and according to the retention time and characteristic UV spectrum of the corresponding ingredients of the standard and Radix Paeoniae Alba Decoction, the paeoniflorin ingredient was separated well in <4 min. The paeoniflorin component was detected in the plasma of healthy SD rats, 30 min following Radix Paeoniae Alba gavage. Fig. 1 shows typical chromatograms of the paeoniflorin standard, Radix Paeoniae Alba decoction and a rat plasma sample following the oral administration of Radix Paeoniae Alba, as well as a blank plasma sample.

### Determination of a suitable gavage dose of paeoniflorin for absorption

The serum IL-1β and TNF-α levels in the model and PF groups were greatly increased compared with those of the normal group. Among the low, medium and high dose paeoniflorin groups, the IL-1β and TNF-α serum levels of the medium dose group were the lowest. Table I shows the mean IL-1β and TNF-α serum levels in each group.

### Effect of paeoniflorin on rats with CIA

**Figure 2:**
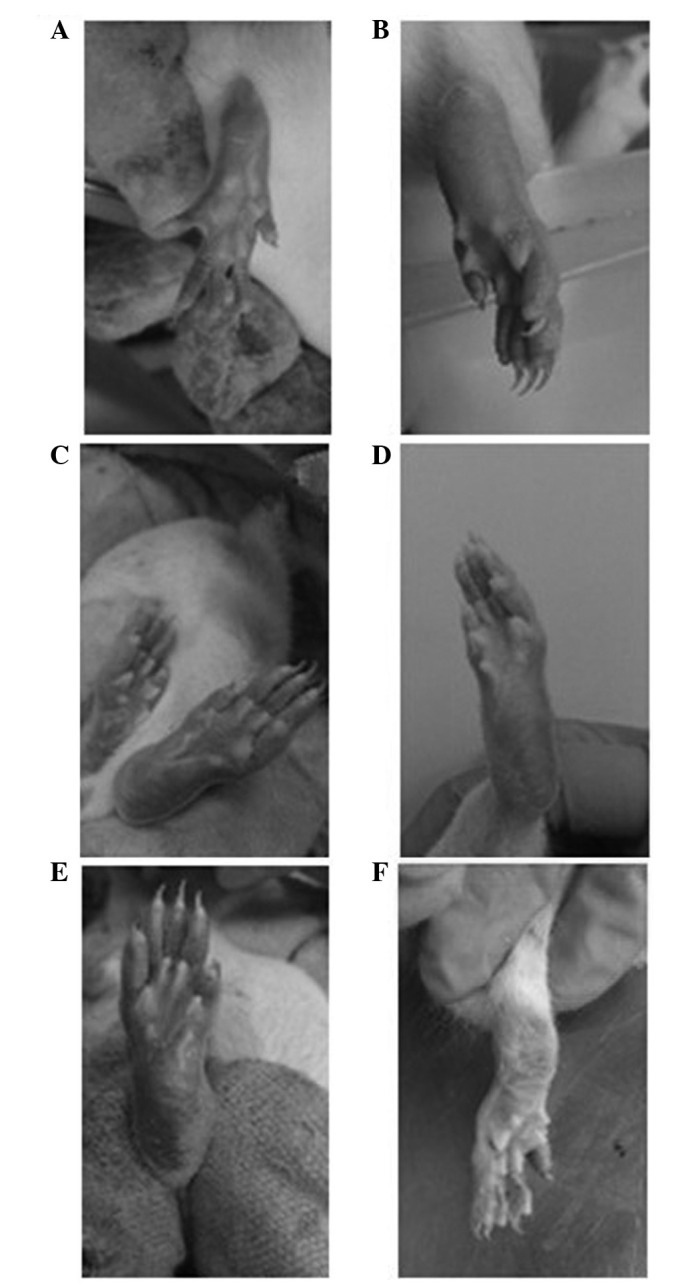
General observations of the rats following immunization. (A) Normal; (B) model (14 days); (C) model (28 days); (D) PF (28 days); (E) model (42 days); and (F) PF (42 days) groups. PF, paeoniflorin.

**Figure 3:**
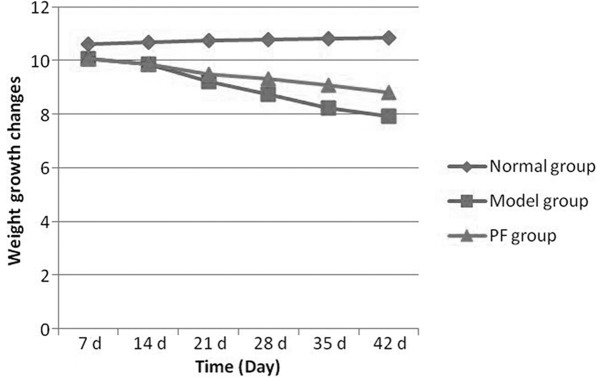
Weight growth changes of the rats (g). PF, paeoniflorin.

Certain rats had joint swelling and ecchymosis 5–7 days following the initial immunization. Following reimmunization on day 7, the joint disease worsened. On day 14, toe joint swelling also worsened and the surface skin of specific joints was shiny and hyperemia was present. On day 42, certain rats had difficulty in weight bearing and movement. During the disease process, rats presented low spirits, somnolence, slow actions, reduced dietary intake and dry hair in the later stages. By evaluating the model rats 2 weeks following immunization, the successful replication rate of the CIA model reached 90%. The rate of weight increase of the normal group did not differ from that prior to modeling. However, 7 days following immunization, the rate of weight increase of the model group rats began to reduce compared with that of the normal group. Following 2 weeks of paeoniflorin treatment, the joint swelling and ecchymosis in the rats with CIA was eased compared with that of the model group. In addition, after 4 weeks, the joint swelling gradually eased, the shiny and hyperemic skin reduced in area, the food intake and activity improved and the hair became shiny (Fig. 2). On days 35 and 42, the weight growth rate of the PF group was more marked than that of the model group. Fig. 3 shows the weight growth changes of the rats in each group.

### Comparison of AI integrals

**Figure 4:**
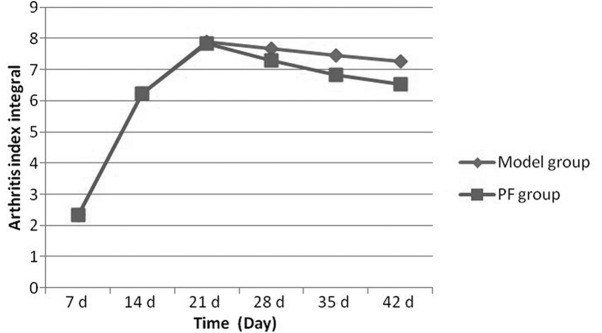
Arthritis index integral of the rats. PF, paeoniflorin.

Signs of arthritis in the rats occurred between 5–7 days after the initial immunization. The AI integral of the model group increased compared with that of the normal group. With the extension in immunization time, the AI integral of the model and PF groups greatly increased and peaked at day 21. However, the AI integral of the PF group was lower than that of the model group. On day 28, the AI integral of the model and PF groups began to decrease and the reduction was more marked in the PF group than in the model group. On day 35 the difference between the model and PF groups was significant. Fig. 4 shows the AI integrals at different time points.

### Measurement of paw swelling

**Figure 5:**
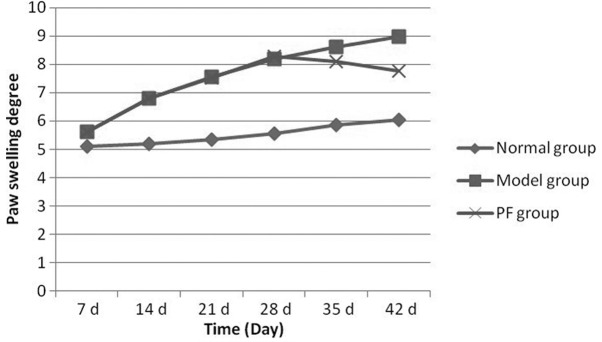
Paw swelling degree of the rats (mm). PF, paeoniflorin.

In the first week of immunization, the foot swelling in the rats was significant. The swelling in the model group was significantly different compared with that in the PF group. With the extension in immunization time, foot swelling in the PF group eased compared with that in the model group. In week 3 of treatment (week 5 of immunization) the difference between the PF and model groups was significant. Fig. 5 shows paw swelling in the rats of each group.

### Analysis of joint X-rays

**Figure 6:**
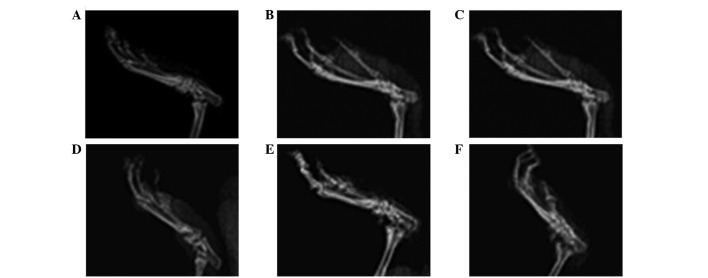
Joint X-ray radiography of the rats from the (A) normal group, (B) model group (14 days), (C) model group (28 days), (D) PF group (28 days), (E) model group (42 days) and (F) PF group (42 days). PF, paeoniflorin.

Normal rats had no swelling of the ankle soft tissue, no bone destruction, an intact structure and clear foot joint space. The X-rays at 2 weeks following immunization (14±2 days) show that the model group rats had tissue swelling around the ankle joint. With the extension of immunization time, the foot joint space was obscured, with narrowing and fusion. In the model group, the first bone change occurred in week 4 (26±4 days) following initial immunization. On day 42, certain areas of joint soft tissue remained moderately swollen and bone destruction was evident. The tissue swelling around the joints in rats with arthritis in the PF group was reduced compared with that in the model group, and the degree of bone destruction was greatly reduced. Fig. 6 shows X-ray images of the joints of rats from each group.

### Histopathological observations

**Figure 7:**
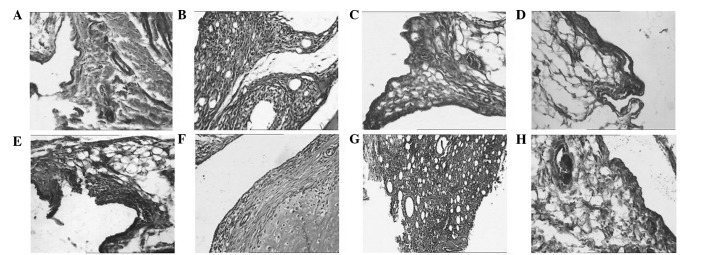
Rat synovial tissue with hematoxylin and eosin staining in the (A) normal group (14 days), (B) model group (14 days), (C) normal group (28 days), (D) normal group (42 days), (E) model group (28 days), (F) PF group (28 days), (G) model group (42 days) and (H) PF group (42 days). PF, paeoniflorin. Magnification, ×100.

The synovial tissue of normal rats had no significant abnormalities 14 days following the initial immunization, whereas that of the model rats exhibited inflammatory cell infiltration. With the extension of immunization time, large amounts of pannus were generated on day 28 and vascular proliferation and synovial thickening occurred at day 42. The deterioration of arthritis caused an increase in the number of new vessels, vascular dilatation and congestion with large quantities of infiltrating lymphocytes. With regard to the PF group, on day 28, a small number of new vessels and inflammatory cell infiltration in the synovial tissue were observed and the synovium had thickened a little. With the extension of the treatment time, on day 42 the number of new vessels was reduced and the inflammation had eased. Fig. 7 shows H&E staining of the synovial tissue in rats from each group.

### Levels of inflammatory cytokines in rat serum

**Figure 8:**
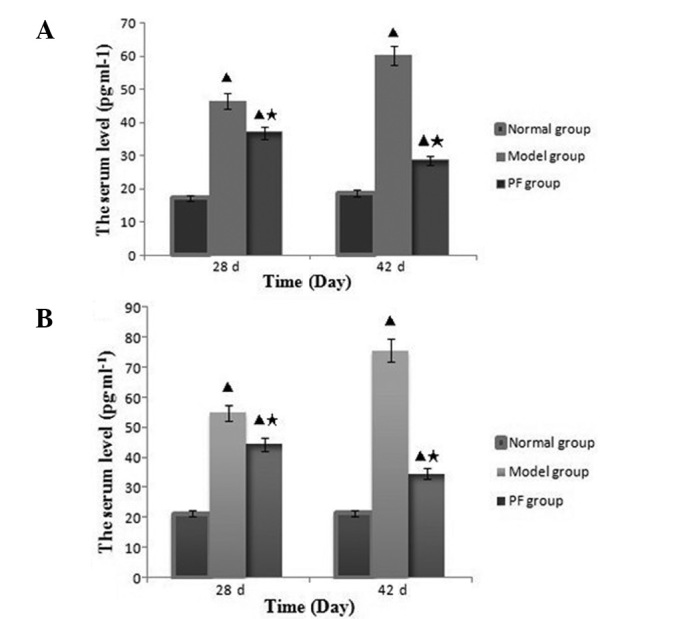
Serum levels of (A) IL-1β and (B) TNF-α in rats at 28 and 42 days following immunization. Data are presented as the mean ± SD. ^▲^P<0.01, vs. the normal group;^★^P<0.01, vs. the model group. IL-1β, interleukin-1β; TNF-α, tumor necrosis factor-α; PF, paeoniflorin.

On day 28 following immunization (14 days following treatment), the IL-1β and TNF-α serum levels of the model and PF groups were markedly increased compared with those of the normal group. However, at this time, the serum IL-1β and TNF-α levels in the PF group were significantly decreased compared with those in the model group (P<0.01). On day 42, the serum IL-1β and TNF-α levels in the model group had increased further, while those of the PF group had decreased; the differences in the IL-1β and TNF-α levels between the groups of rats were significant at 28 and 42 days (P<0.01). Fig. 8 shows the serum IL-1β and TNF-α levels in the normal, model and PF groups.

## Discussion

The development of modern analytical instruments and technology indicates broad prospects for the determination of the compositions of TCMs. In 2004, Waters Corporation introduced the world’s first UPLC instrument. This revolutionary technology eliminated the limitations of traditional chromatographic analysis systems, solving the problem of pressure proofing the whole system. In addition, UPLC has a high column efficiency, which increases the degree of separation, speed of analysis and detection sensitivity to unprecedented degrees (17,18). UPLC not only improves the quality of analysis results, it also reduces the running time and the quantity of solvent ≥10-fold, thereby saving considerable time, money, energy and space, and improving the efficiency overall (19,20). As a result, UPLC plays a vital role in the pharmaceutical and biomedical fields (21,22). The combined UPLC-PDA detector is a convenient and widely used technology utilized for the routine detection of a variety of components of plant-based drugs. UPLC is now considered to be one of the most promising analysis techniques in the chromatographic field. For the separation and determination of the effective components of TCMs, UPLC has certain advantages compared with conventional HPLC, including high speed, high sensitivity, high resolution, time saving and reduced consumption (23). In the present study, the detection of paeoniflorin in a Radix Paeoniae Alba decoction by a UPLC-PDA method was completed in <4 min. This separation time is lower than the previously reported time of ~4 min (24). The experiments in which Radix Paeoniae Alba decoction was administered to rats indicate that paeoniflorin is an active ingredient of the decoction that is absorbed by rat plasma. To the best of our knowledge, this has not been reported previously.

RA in TCM belongs to the Chinese medicine ‘arthralgia category’. TCM theory considers RA to be an asthenic disease, including deficiency and excess mixing as the main manifestations (25). TCM has a long history and rich resources in the treatment of RA. In previous years, a number of studies have shown that specific TCM components are useful in the treatment of RA (26). These components have been reported to downregulate cytokine expression, alleviate inflammation in RA, inhibit fibroblast-like synoviocytes (FLS) proliferation and promote apoptosis, thereby reducing synovial hyperplasia, inhibiting human chondrocyte and cartilage degradation and preventing joint bone destruction; multiple targets and pathways in RA are affected (26). The analysis of the effects of TCM components also provides a theoretical basis for studies of the mechanisms of Chinese herbal compounds, which contain these effective components.

BZXD is a TCM that was formulated by our department. It has been used clinically for a number of years to treat RA and has good clinical efficacy (2). Previous studies have shown that BZXD is involved in the regulation of the immune system, the inhibition of inflammatory cytokines, synovial angiogenesis and bone destruction, and the adjustment of the abnormal expression of genes and proteins (3–8). The chemical constituents of TCMs are complex and the majority are not absorbed by the body following oral administration. The functioning component is the material that is absorbed into the blood, i.e., the transitional ingredients in the serum. A previous study on the serum pharmacochemistry of TCM considered that only the ingredients that are absorbed into the blood are the effective components (27). The current study analyzed rat plasma following the oral administration of Radix Paeoniae Alba decoction and demonstrated that paeoniflorin is an active ingredient that is absorbed by rat plasma. These observations indicate that paeoniflorin is likely to be one of the active substances of BZXD and may play a vital role in the treatment of RA. At present, studies concerning the measurement of the paeoniflorin content of plasma are limited.

Radix Paeoniae Alba is an important component of BZXD. It has a number of functions, including nourishing the blood and liver, reducing pain and astringing Yin sweat (9). The effective components of Radix Paeoniae Alba mainly include a series of aminoglycoside substances, including paeoniflorin, hydroxy-paeoniflorin, peony glucoside, albiflorin and benzoylpaeoniflorin, which are collectively referred to as TGP. In Radix Paeoniae Alba, paeoniflorin accounts for >90% of the total glucosides and is the main effective component. Numerous studies on the pharmacological effect of paeoniflorin have been performed, which have revealed that paeoniflorin functions as an anti-free radical agent, exhibiting antineurotoxic properties and inhibiting intracellular calcium overload (28). *In vivo* studies have shown that paeoniflorin has numerous biological effects, including blood viscosity reduction, the inhibition of platelet aggregation, the dilation of blood vessels and improvement of the microcirculation and functions as an antioxidant and anticonvulsant with limited side-effects (29,30,31). Studies concerning the pharmacological effects of paeoniflorin have mainly focused on the effects it has on the nervous system (32,33). With regard to the role of paeoniflorin in RA, studies have shown that paeoniflorin has significant inhibitory effects in rats with AA and rats with CIA and secondary paw swelling, markedly improves the pain reaction associated with polyarthritis and the paw of secondary arthritis, and exhibits a regulatory effect on imbalanced inflammatory cytokine secretion (34–36). However, there are also a small number of studies indicating that paeoniflorin inhibits the bone erosion associated with the pathological process of RA. In the present study, rats with CIA were treated with paeoniflorin, which led to significant reductions in the swelling, joint AI and the levels of inflammatory cytokines, IL-1β and TNF-α. In addition, the inflammatory reaction in rats with CIA was inhibited. IL-1β and TNF-α play key roles in the synovitis and cartilage and bone destruction of RA (37,38). IL-1β promotes osteoblast expression and osteoclast absorption (4). IL-1β is a major cytokine that amplifies the inflammatory response of RA and transforms it into a damaging reaction. TNF-α is a proinflammatory cytokine that is important for the pathogenesis of RA; it is involved in the pathogenic mechanism of RA, including endothelial cell activation, cytokine induction, white blood cell aggregation, activation of osteoclasts and the destruction of cartilage and bone cells (39) and leads to a continued inflammatory reaction and the progressive destruction of cartilage and bone.

The results of the present study show that paeoniflorin reduced the levels of the inflammatory cytokine IL-1β and TNF-α, and reduced the soft tissue swelling of the joints of the arthritic rats. The degree of joint bone destruction decreased significantly compared with that in the model group, indicating that paeoniflorin may inhibit bone erosion in RA. These observations provide a scientific basis for the use of paeoniflorin in the treatment of RA, laying the foundations for future studies into the mechanism of action of Radix Paeoniae Alba and providing a new method for the further study of the biologically active ingredients of the efficacy of BZXD. This method, which combines the analysis of the effective components of a TCM with UPLC-PDA and with intervention in animal models, is likely to provide a new approach for the study of effective TCM components in the future. In addition, it may aid the identification of new drugs for the treatment of RA.

## Figures and Tables

**Table I:** Serum levels of TNF-α and IL-1β in rats (pg/ml, mean ± SD).

Groups	IL-1β	TNF-α
Normal	19.51±1.09	21.92±1.66
Model	60.41±2.56^a^	75.21±1.86^a^
Low dose PF	34.77±1.72^a^–^c^	39.44±2.12^a^–^c^
Middle dose PF	24.72±1.98^a^,^b^	31.38±2.40^a^,^b^
High dose PF	31.77±1.97^a^–^c^	36.76±1.92^a^–^c^

Values presented are the mean ± SD.

a P<0.01 vs. the normal group;

b P<0.01 vs. the model group;

c P<0.01 vs. the middle dose PF group.

PF, peoniflorin; IL-1β, interleukin-1β; TNF-α, tumor necrosis factor-α.
